# Synergistic effects of combined treatment with histone deacetylase inhibitor suberoylanilide hydroxamic acid and TRAIL on human breast cancer cells

**DOI:** 10.1038/srep28004

**Published:** 2016-06-13

**Authors:** Weiqiang Zhou, Xiuyan Feng, Han Han, Shanchun Guo, Guangdi Wang

**Affiliations:** 1 Key Laboratory of Environmental Pollution and Microecology of Liaoning Province, Shenyang Medical College, No. 146 North Huanghe St, Huanggu Dis, Shenyang City, Liaoning Pro 110034, P. R. China; 2 The Second Affiliated Hospital of Shenyang Medical College, No. 20 North 9th St, Heping Dis, Shenyang City, Liaoning Pro 110002, P. R. China; 3 RCMI Cancer Research Center, Xavier University of Louisiana, New Orleans, LA 70125, USA; 4 Department of Chemistry, Xavier University of Louisiana, New Orleans, LA 70125, USA

## Abstract

Previous studies showed that either histone deacetylase (HDAC) inhibitors or tumor necrosis factor-related apoptosis-inducing ligand (TRAIL) can induce apoptosis in tumor cells including breast cancer. However, the underling mechanisms of combining HDAC inhibitors with TRAIL in the treatment of breast cancer are poorly understood. In this study, we determined the ability of SAHA and TRAIL as single agents or in combination to inhibit the growth and survival of MCF-7 and MDA-MB-231 breast cancer cells. Our results demonstrate that the distinct effects of SAHA or TRAIL individually and in combination on the proliferation, cell viability, apoptosis, cell cycle distribution, and morphological changes of MDA-MB-231 and MCF-7 cells. We further determined the different effects of SAHA or TRAIL alone and combining SAHA with TRAIL on the expression of a number of apoptosis-related molecules, cell cycle, growth factors and their receptors in cancer cells. Our results demonstrated that the combinatorial treatment of SAHA and TRAIL may target multiple pathways and serve as an effective therapeutic strategy against breast cancer. An improved understanding of the molecular mechanisms may facilitate either SAHA or TRAIL targeted use and the selection of suitable combinations.

Breast cancer is the most common malignant disease in women worldwide with 1.67 million new cases diagnosed and 522,000 breast cancer-related deaths in 2012^1^. Clinically, estrogen receptor (ER), along with progesterone receptor (PgR) and human epidermal growth factor receptor 2 (Her2) expression status are essential molecular markers for the assessment of adjuvant treatment options and prognosis for breast cancer patients. According to ER phenotypic differences, breast cancer can be divided into two types: ER-positive and ER-negative. Approximately two thirds of all breast cancer patients are ER-positive, showing less tissue necrosis, flexibility, low lymphatic invasion, sensitive to anti-estrogen therapy with clinical response rate 50–60%^2^,^3^. Patients of ER-negative breast cancer often present high degree of malignancy, aggression and poor prognosis despite initial responsiveness to chemotherapy^4^,^5^.

Epigenetic modification of gene expression plays an important role in carcinogenesis. Emerging data indicate that epigenetic changes affect the ER status in breast cancer with acquired resistance^6^,^7^,^8^. Histone deacetylases (HDAC) are chromatin modifiers that lead to epigenetic changes in the regulation of steroid hormone receptor mediated cell signaling, and their inhibition potentiates the therapeutic efficacy of anti-estrogens^9^,^10^,^11^,^12^. Suberoylanilide hydroxamic acid (SAHA, vorinostat) is a pan HDAC inhibitor that depresses HDAC activity by acting on all 11 known human class I and class II HDACs^13^. SAHA dramatically changes cellular acetylation patterns and causes growth arrest and death in a broad variety of transformed cells, both *in vitro* and in animal tumor models^13^,^14^. SAHA is indicated for the treatment of cutaneous T cell lymphoma (CTCL) with a large number of ongoing clinical trials to evaluate its utility in treating various solid tumors. Studies have shown that SAHA can induce apoptosis and growth arrest in breast cancer cell lines including MCF-7, MDA-MB-231, MDA-MB-435, MDA-MB-468, and SKBr-3^15^,^16^,^17^,^18^,^19^. On the other hand, due to rapid hepatic glucuronidation, SAHA has a short half-life of 2 hrs, making it difficult to provide the level of drug exposure necessary for durable therapeutic efficacy on solid tumors. Adverse side effects, which become more severe at escalated doses, and intrinsic and acquired resistance to vorinostat also present significant clinical challenges^20^,^21^.

Tumor necrosis factor-related apoptosis-inducing ligand (TRAIL) has been recognized as having a key role in body’s natural defense mechanism and in inducing apoptosis in a variety of tumor cells, but its clinical utility has been limitated^22^,^23^,^24^,^25^. TRAIL mediated apoptosis is initiated by the binding of two agonistic death receptors, DR4 (TRAIL-RI) and DR5 (TRAIL-RII) in a p53-independent manner^26^,^27^,^28^. Conversely, TRAIL activity can be specifically inhibited by two decoy receptors, DcR1 (TRAIL-R3, LIT or TRID) or DcR2 (TRAIL-R4 or TRUNDD) thereby blocking its signaling of cell death^29^. TRAIL can also bind to osteoprotegerin (OPG), a soluble receptor for TRAIL, to attenuate apoptosis^30^,^31^. TRAIL preferentially induces apoptosis in tumor cell lines that lack DcR1, DcR2, but not in normal cells which express DcR1, DcR2, suggesting that TRAIL could potentially represent a powerful cancer therapeutic^32^,^33^. In recent years, TRAIL-based combinatorial therapies are emerging paradigms for cancer treatment since synergistic activation of TRAIL-induced apoptosis by chemotherapeutic drugs can generally overcome tumor cell resistance, while monotherapies are often fail. Preclinical studies and clinical trials are introducing promising results, supporting the potential effects of these combined approaches^34^,^35^.

A number of preclinical studies combining HDAC inhibitors with TRAIL have shown synergistic effects in inhibition of proliferation and induction of apoptosis in tumor cells^36^. SAHA was reported to induce expression of TRAIL by directly activating its promoter and triggering TRAIL-mediated apoptosis in acute myeloid leukemia cells^37^. Antisense ablation of TRAIL in the sensitive HL60 cells significantly reduced SAHA-mediated apoptotic and cytotoxic effects, indicating that TRAIL signaling pathway was important for SAHA pharmacological action^38^. In breast cancer cells, several HDAC inhibitors have been shown to enhance TRAIL-mediated apoptosis^39^,^40^. For example, SAHA can sensitize TRAIL-resistant breast cancer cells^17^,^41^. However, the underlying mechanisms of combining HDAC inhibitors with TRAIL in the treatment of breast cancer are poorly understood. The purpose of this study was to determine the ability of combining SAHA with TRAIL to selectively target the breast cancer cells, assessed by their combined effects on the growth and survival of a representative panel of breast cancer cells. We also sought to characterize the effects of combining SAHA with TRAIL on the regulation of breast cancer genes, related signaling pathways, and morphology.

## Results

### Real-time monitoring of the effects of SAHA on the cell index (CI) of MDA-MB-231 and MCF-7 cells

**Figure 1:**
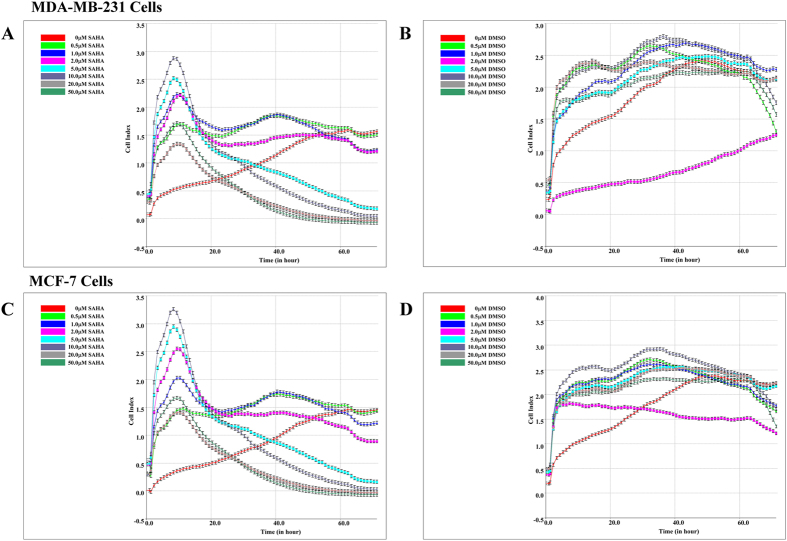
Real-time monitoring of the effects of SAHA on the cell index (CI). 1.0 × 10^4^/well of breast cancer cells was added into E-plate 16 in duplicates, and the cells were incubated with medium containing SAHA (0, 0.5, 1, 2, 5, 10, 20 and 50 μM). The same concentrations of DMSO were used as control. xCELLigence Real-time Cell Analyzer (RTCA) DP system was used to monitor the value changes of cell index. (**A**) MDA-MB-231 cell, treated with SAHA. (**B**) MDA-MB-231 cell, treated with DMSO. (**C**) MCF-7 cell, treated with SAHA. (**D**) MCF-7 cell, treated with DMSO.

Preliminary experiments were performed to evaluate the effects exerted by SAHA in MDA-MB-231 and MCF-7 cells. The cells were seeded in 96-well plates, incubated with 0–50 μM SAHA, then evaluated through real-time monitoring of cell growth by xCELLigence Real-time Cell Analyzer (RTCA) DP system. Since the changes of cell index (CI) directly correlate to cell proliferation, cytotoxicity, adhesion, and viability, the changes of CI can directly reflect the effects of drugs. In the control group without SAHA treatment, both MDA-MB-231 and MCF-7 cells showed robust growth; CI continued to increase over time and reached a plateau at 48 h (Fig. 1A,C). In contrast, in the group with SAHA treatment, both cell lines showed a time- and dose-dependent CI values. Initially treatment of SAHA induced a remarkable increase of CI values which peaked at around 10 hours. After the spike, SAHA decreased CI values of both cell lines over progression of time. Large doses of SAHA (20–50 μM) showed significant effects even at 24 h compared to lower doses of SAHA (0.5 μM–2.0 μM) that showed minimal effects on the cell condition. When the dose of SAHA reached 5.0 μM, both cell lines displayed statistically significant decrease in CI values compared to the control group. We also examined the effects of DMSO on the growth of MDA-MB-231 and MCF-7 cells. DMSO did not show significant cytotoxicity, and CI values continued to increase in both cell lines, although CI curves appear different from those of untreated cells (Fig. 1B,D).

### Real-time monitoring of the effect of SAHA/TRAIL combination on breast cancer cell proliferation

**Figure 2:**
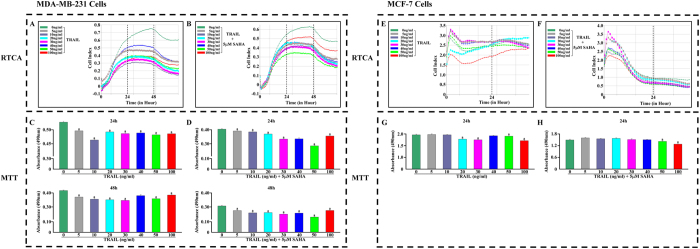
Real-time monitoring the cell index (CI) and cell proliferation assays. 1.0 × 10^4^/well of breast cancer cells was added into E-plate 16 in duplicates, and the cells were incubated with medium containing 5 μM SAHA and TRAIL (0, 5, 10, 20, 30, 40, 50, 100 ng/ml). The same concentrations of DMSO were used as control. xCELLigence Real-time Cell Analyzer (RTCA) DP system was used to monitor the value changes of cell index. (**A**) MDA-MB-231 cell, treated with TRAIL. (**B**) MDA-MB-231 cell, treated with SAHA and TRAIL. (**E**) MCF-7 cell, treated with TRAIL. (**F**) MCF-7 cell, treated with SAHA and TRAIL. For 24 and 48 hours incubation, the culture supernatants were used to assess drug dose-response effects using the CellTiter 96 Aqueous One Solution Cell Proliferation Assay. The spectrophotometric absorbance of each sample was measured at 490 nm using TECAN spectora. (**C**) MDA-MB-231 cell, treated with TRAIL. (**D**) MDA-MB-231 cell, treated with SAHA and TRAIL. For 24 hours incubation, (**G**) MCF-7 cell, treated with TRAIL. H. MCF-7 cell, treated with SAHA and TRAIL. (a) *p* < 0.05 when comparing levels of absorbance (490 nm) to control. Data (mean ± standard error) representative results derived from a minimum of 3 independent experiments.

In order to investigate the effect of TRAIL on breast cancer cell proliferation, MDA-MB-231 or MCF-7 cells was individually co-cultured with 0–100 ng/ml of varying concentrations of TRAIL in xCELLigence RTCA system. The results revealed that the effects of TRAIL on MDA-MB-231 and MCF-7 cell lines are entirely different. The inhibitory effect of TRAIL, from low to high-dose on MDA-MB-231, is obvious. In contrast, MCF-7 cell line is insensitive to TRAIL except at a high concentration, 100 ng/ml, of TRAIL when more pronounced growth inhibition effect was observed (Fig. 2A,E).

We next determined the combined effects of 0–100 ng/ml TRAIL and 5 μM SAHA on MDA-MB-231 and MCF-7 cells. Although the combined effects on MDA-MB-231 cells did not show dose-dependency, the CI dropped significantly. TRAIL at 50 ng/ml concentration, reaction time at 48 h, showed the strongest results in the combined trial (Fig. 2B). The combined effect of SAHA and TRAIL on MCF-7 cells showed more pronounced growth inhibition. Even at 24 h, the CI fell to the bottom of the baseline (Fig. 2F).

The treatments of TRAIL alone and combined TRAIL with SAHA on MDA-MB-231 cells showed similar inhibiting effect when comparing the cell response curves. However, the effects of combined TRAIL and SAHA treatment on MCF-7 cells resulted in a sharp decline of CI, indicating that the combination of two drugs may increase the sensitivity of MCF-7 cells.

We further confirmed the results using the CellTiter 96 Aqueous One Solution Cell Proliferation Assay (Fig. 2C,D,G,H). Finally, we determined the optimal treatment time and concentration of combined TRAIL and SAHA where maximum effect is observed. We found that, for MDA-MB-231 cells, the combination of 50 ng/ml TRAIL and 5 μM SAHA for 48 h treatment yielded the most effective inhibition of cell growth. For MCF-7 cells, 100 ng/ml TRAIL and 5 μM SAHA for 24 hrs of treatment was the ideal combination.

### The effect of SAHA/TRAIL combination on cell viability and apoptosis of cancer cells

**Figure 3:**
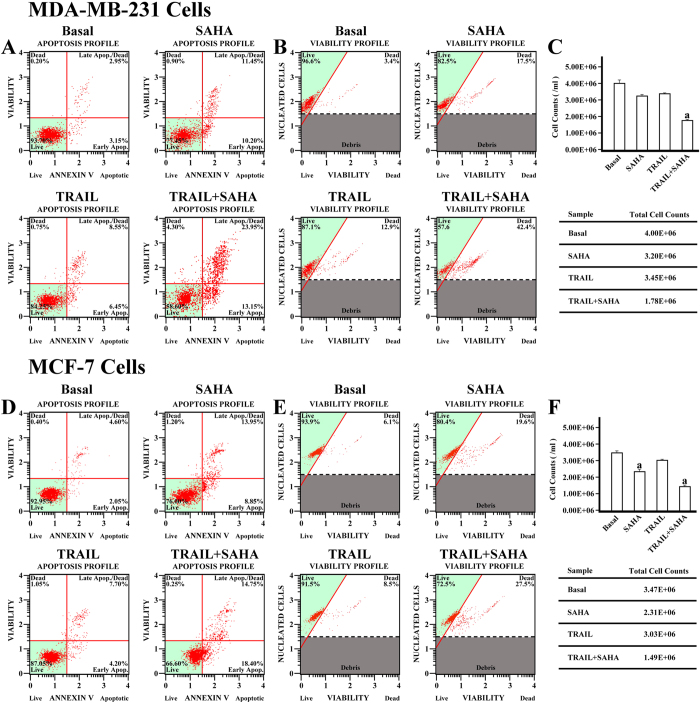
Cell Viability and Apoptosis assay for MDA-MB-231 and MCF-7 cell. MDA-MB-231 cell was plated in 6 well plates at a density of 5 × 10^5^ cells per well. The cells were incubated in complete culture medium containing 5 μM SAHA or combining with 50 ng/ml TRAIL for 48 hours. For the apoptotic assay, 1 × 10^6^ of cells were transferred in suspension to a new tube and incubated with 100 μl of Muse Annexin V & Dead Cell reagent (Millipore) for 20 minutes at room temperature. The apoptosis was determined by Muse Cell Analyzer (Millipore) and the statistics were shown the percentages of the cells represented by alive, apoptosis and dead population (**A**). Cell viability assay was performed by the Muse Count & Viability reagent (Millipore) following the manufacturer’s protocols. Following trypsinization, 2 × 10^5^ of harvested cells were added with 450 ul Count & Viability reagent (Millipore). The results were obtained with Muse Count & Viability software module and the statistics were shown the percentage of viable cells ((**B**) cell viability, (**C**) cell count). MCF-7 cell was also plated in 6 well plates at a density of 5 × 10^5^ cells per well. The cells were incubated in complete culture medium containing 5 μM SAHA or combining with 100 ng/ml TRAIL for 24 hours. For the apoptotic assay, same as MDA-MB-231. The apoptosis was determined by Muse Cell Analyzer (Millipore) and the statistics were shown the percentages of the cells represented by alive, apoptosis and dead population (**D**). Cell viability assay was performed by the Muse Count & Viability reagent (Millipore) following the manufacturer’s protocols. Following trypsinization, 2 × 10^5^ of harvested cells (50 ul cell suspension) were added with 450 ul Count & Viability reagent (Millipore). The results were obtained with Muse Count & Viability software module and the statistics were shown the percentage of viable cells ((**E**) cell viability, (**F**) cell count). (a) *p* < 0.05 when comparing cell numbers to control. Data (mean ± standard error) representative results derived from a minimum of 3 independent experiments.

To further confirm the effects of SAHA and TRAIL on breast cancer cell proliferation, we determined the cell viability and apoptosis in MDA-MB-231 and MCF-7 cell lines. In comparison with DMSO control treatment, both cell viability and cell number decreased in MDA-MB-231 and MCF-7 cells after SAHA treatments. TRAIL alone was slightly less effective than SAHA’ effect in inhibiting growth of both cell lines. However, combined TRAIL and SAHA treatment induced dramatic decreases in cell viability and cell number of both MDA-MB-231 and MCF-7 cells with over 50% reduction in the cell number (Fig. 3B,C,E,F). As expected, in comparison with DMSO control treatments, the apoptotic cells increased in MDA-MB-231 and MCF-7 cells after the SAHA treatment. TRAIL alone only showed slight increase in apoptotic cells in the two cell lines. However, the apoptotic cells dramatically increased in MDA-MB-231 and MCF-7 cells after combining TRAIL and SAHA treatment; the apoptotic rate reached 13.15% in the early stage and 23.95% in the late stage in MDA-MB-231. The apoptotic rate reached 18.40% in the early stage and 14.75% in the late stage in MCF-7 cells (Fig. 3A,D).

### The effect of SAHA/TRAIL combination on cancer cell cycle distribution

**Figure 4:**
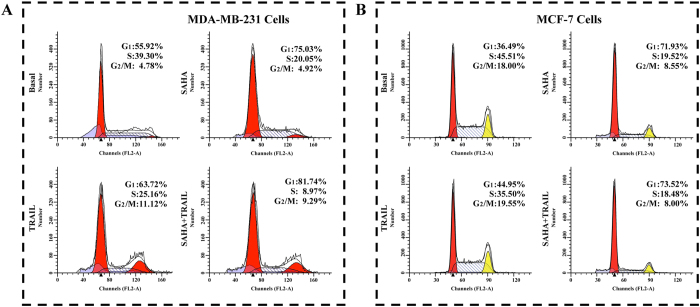
Cell Cycle assay. 5 × 10^5^ of MDA-MB-231 or MCF-7 cells were treated with 5 μM SAHA or combining with 100 ng/ml TRAIL 24 hours as described above. After treatment with 5 μM SAHA or combining with 100 ng/ml TRAIL as described above. The harvested cells were centrifuged at 300 g for 5 minutes. While mixing and resuspending cells, cells were slowly added with 1ml of ice cold 70% ethanol and incubated overnight at −20 °C. 10 μl PI was added to the cell suspension. Samples were filtered through 200-mesh filters and then analyzed on BD FACS Calibur flow cytometer. The DNA content of the cells was analyzed for the percentage of cells in G_1_, S and G_2_/M phase. (**A**) MDA-MB-231 cell, (**B**) MCF-7 cell.

Next, we determined the effects of SAHA and TRAIL on the changes of cell cycle of breast cancer cells. The results revealed that the effects of SAHA and TRAIL on cell cycle of MDA-MB-231, MCF-7 cell lines are different. For MDA-MB-231 treated with SAHA, the cells in G0/G1 phase increased from 55.92% to 75.03%, the cells in S phase decreased from 39.3% to 20.05%. No significant changes were noted in the G2/M phase of the MDA-MB-231 cells treated with SAHA. Treatment of TRIAL slightly increased cells in G0/G1phase, reduced cells in S phase from 39.3% to 25.16%, and increased G2/M cells from 4.78% to 11.12%. Combination treatment of TRAIL and SAHA reduced the cells in S phase from 39.3% to 9.29%, while 81.74% of total cells were confined in G0/G1 phase (Fig. 4A). For MCF-7 cells treated with SAHA, the G0/G1 phase cells increased from 36.49% to 71.93%, the cells in S phase decreased from 45.51% to 19.52% and the cells in G2/M decreased from 18.00% to 8.55%. For the treatment of TRAIL, there were slightly increased cells in G1/S phase, slightly decreased cells in S phase and no significant changes of cell numbers in G2/M. For combined SAHA and TRAIL treatment, the cell numbers showed slightly more changes in three different phases than SAHA treatment alone (Fig. 4B).

### The effect of SAHA/TRAIL combination on morphological changes of cancer cells

**Figure 5:**
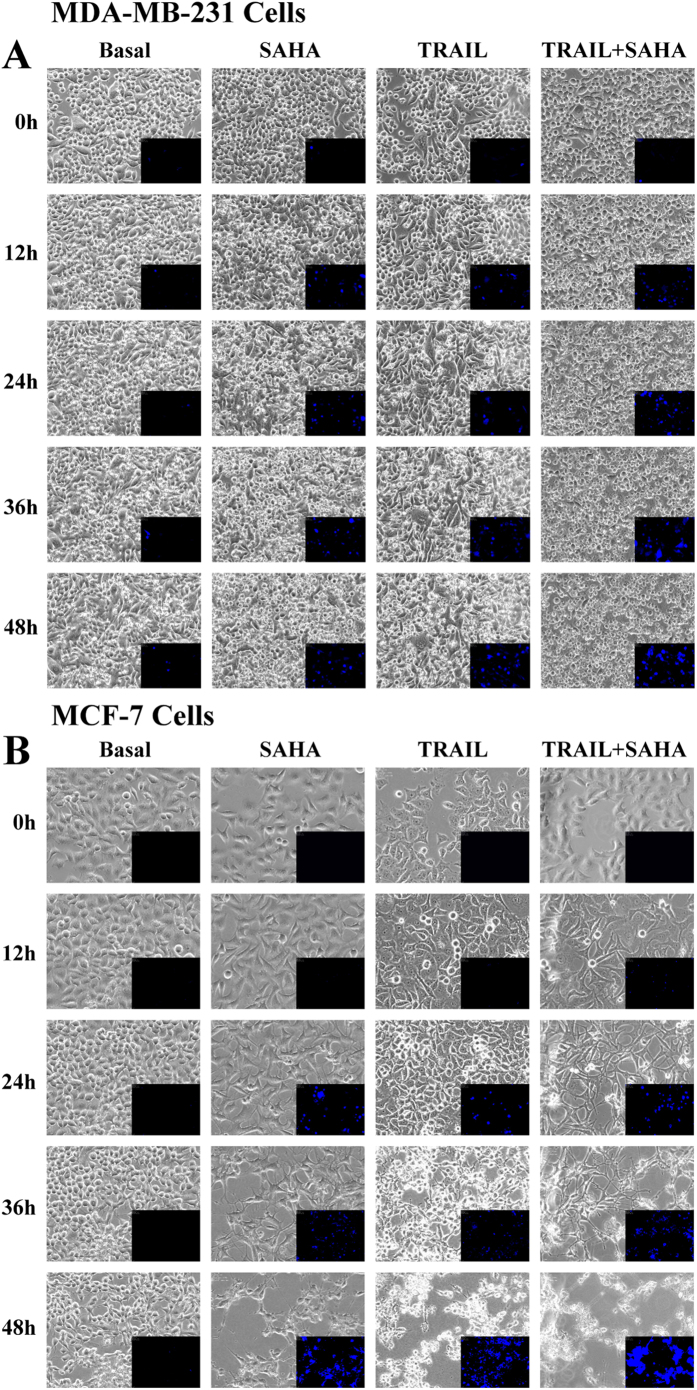
Time-lapse live cell imaging acquisition. (**A**) A density of 5 × 10^5^ MDA-MB-231 cells was loaded in the each well of 4-chamber system. SAHA, TRAIL alone and combination treatments were initiated on experimental day 3 after starvation and aggregate formation. Phase contrast microscopic images of cells were obtained using the BioStation IM-Q. Image acquisition timing was set to every 12 hour (magnification = 20x) and time points were designated as time 0, 12, 24, 36 and 48, indicating each of the 12 hour imaging intervals. Annexin-V-FLUOS staining kit was used for apoptosis detection with desired concentrations of SAHA and TRAIL for 0, 12, 24, 36 and 48 hours and immediately analyzed by fluorescence microscopy. (**B**) A density of 5 × 10^5^ MCF-7 cells was loaded in the each well of 4-chamber system. SAHA, TRAIL alone and combination treatments were initiated on experimental day 3 after starvation and aggregate formation. Phase contrast microscopic images of cells were obtained using the BioStation IM-Q. Image acquisition timing was set to every 12 hour (magnification = 20x) and time points were designated as time 0, 12, 24, 36 and 48, indicating each of the 12 hour imaging intervals. Annexin-V-FLUOS staining kit was used for apoptosis detection with desired concentrations of SAHA and TRAIL for 0, 12, 24, 36 and 48 hours and immediately analyzed by fluorescence microscopy.

We further examined the morphological changes of cancer cells after SAHA or TRAIL treatments using Nalge-Nunc “Lab-Tek^®^” coverglass system and BioStation IM-Q. The cells grew well and occupied to 90% of the full plate after 48 hrs in the control treatment of DMSO. We observed distinct morphological changes of cancer cells after TRAIL or SAHA treatment alone. For MDA-MB-231, some cells showed apoptotic morphology characterized by cell rounding and shrinkage, appearance of membrane bubbles and several condensed or fragmented nuclei, which were further measured with Annexin V positivity even at 12 hrs after SAHA treatments. Apoptotic cells were seen at 24 hrs after TRAIL treatment. In contrast, apoptotic cells were more evident in reaching 50% of the total cells at 24 hrs after combined treatment of TRAIL and SAHA (Fig. 5A). For MCF-7, more apoptotic cells were seen at 36 hrs after SAHA treatments. Only a small number of apoptotic cells were seen at 24 hrs, but more were evident at 36 hrs after TRAIL treatment. However, apoptotic cells were observed at 12 hrs, and almost all cells were dead at 48 hrs after combined TRAIL and SAHA treatments (Fig. 5B).

### The effects of SAHA and TRAIL on the expression of apoptosis-related molecules in cancer cells

**Figure 6:**
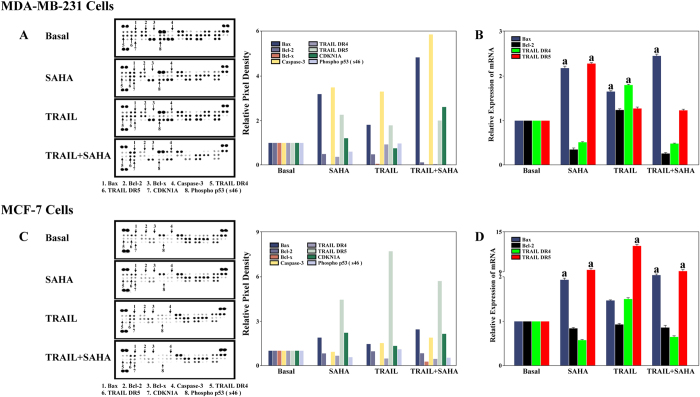
(**A**) Human Apoptosis Antibody Array. Approximately 1 × 10^7^ MDA-MB-231 cells with SAHA and TRAIL treatment were solubilized and 200 ug of prepared cell lysates were detected by apoptosis antibody array. Membrane intensity was acquired using chemiluminescence and pixel densities can be analyzed using Gelpro Analyzer software. (**B**) Real-time PCR. 1 × 10^6^ of MDA-MB-231 were treated with 5 μM SAHA or combining with 100 ng/ml TRAIL described as above. Real-time PCR reactions were used to analyze the expression levels of related genes. GAPDH was as an endogenous control. (**C**) Human Apoptosis Antibody Array. Approximately 1 × 10^7^ MCF-7 cells with SAHA and TRAIL treatment were solubilized and 200 ug of prepared cell lysates were detected by apoptosis antibody array. Membrane intensity was acquired using chemiluminescence and pixel densities can be analyzed using Gelpro Analyzer software. (**D**) Real-time PCR. 1 × 10^6^ MCF-7 cells were treated with 5 μM SAHA or combining with 100 ng/ml TRAIL described as above. Real-time PCR reactions were used to analyze the expression levels of related genes. GAPDH was as an endogenous control.

In order to clarify the mechanisms of apoptosis induction by SAHA and TRAIL in breast cancer cells, we determined a number of apoptosis-related protein expression using antibody array. SAHA alone inhibited Bcl-2, Bcl-x and Phospho P53 (s46) expression while inducing DR5 and CDKN1A expression in both cell lines. SAHA alone also significantly induced Caspase-3 expression in MDA-MB-231 but not MCF-7 cells. TRAIL alone significantly induced Bax, Caspase-3 and DR5 expression, and it inhibited Bcl-2, Bcl-x, CDKN1A expression in MDA-MB-231 cells. In MCF-7 cells, TRAIL alone induced DR5 and CDKN1A expression. As expected, combined TRAIL and SAHA treatments significantly induced Bax, DR5 and CDKN1A, and they also significantly inhibited Bcl-2, Bcl-x and Phospho P53 (s46) expression in both of the cell lines. Notably, in comparison with control treatment, SAHA alone or SAHA plus TRAIL induced CDKN1A expression accompanied by decreased Phospho P53 (s46) expression in both MCF-7 and MDA-MB-231 cells. However, TRAIL alone did not show this correlation in neither cell line (Fig. 6A,C). In addition, we observed that combined TRAIL and SAHA treatments apparently increased Bax, Caspase-3 and CDKN1A in MDA-MB-231 cells and only Bax in MCF-7 compared to TRAIL or SAHA alone. We then determined the mRNA expression of apoptosis-related molecules, Bax and Bcl-2, TRAIL related receptors, DR4, DR5 using real time PCR. We found that SAHA dramatically induced DR5, but not DR4 mRNA expression in both MDA-MB-231 and MCF-7 cell lines. SAHA inhibited Bcl-2 mRNA expression in MDA-MB-231 cell line. TRAIL or SAHA alone, as well as combined TRAIL and SAHA treatments significantly induced Bax mRNA expression in both cell lines, and they also inhibited Bcl-2 mRNA expression in MDA-MB-231 cells (Fig. 6B,D).

**Figure 7:**
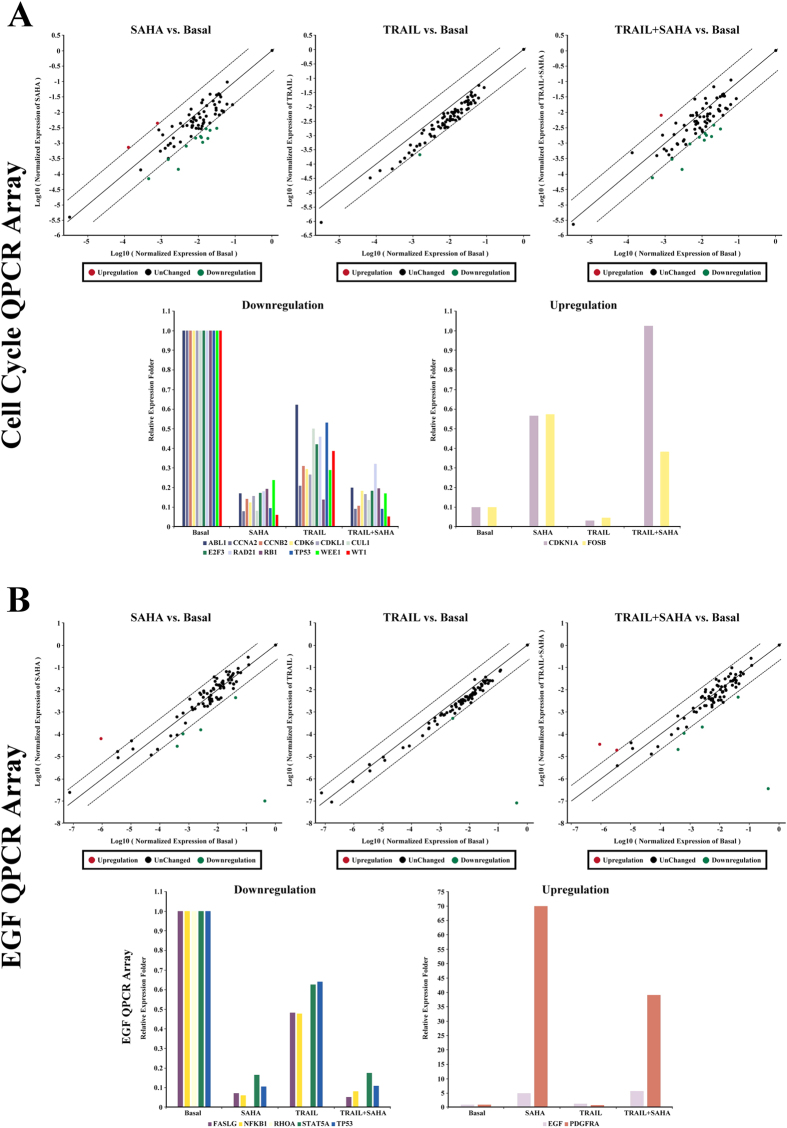
Q-PCR Array. (**A**) Human qPCR arrays for cell cycle related gene were employed to validate the mRNA expression for MDA-MB-231 cells treatment with SAHA and TRAIL. Data normalization was based on correcting all *C*_t_ values for the average *C*_t_ values of GAPDH gene present on the array. Scatter plot and multigroup plot analysis were used respectively to show the data. Three independent biological replicates were performed. (**B**) Human qPCR arrays for EGF/PDGF signaling related gene were employed to validate the mRNA expression for MDA-MB-231 cells treatment with SAHA and TRAIL. Data normalization was based on correcting all *C*_t_ values for the average *C*_t_ values of GAPDH gene present on the array. Scatter plot and multigroup plot analysis were used respectively to show the data. Three independent biological replicates were performed.

**Figure 8:**
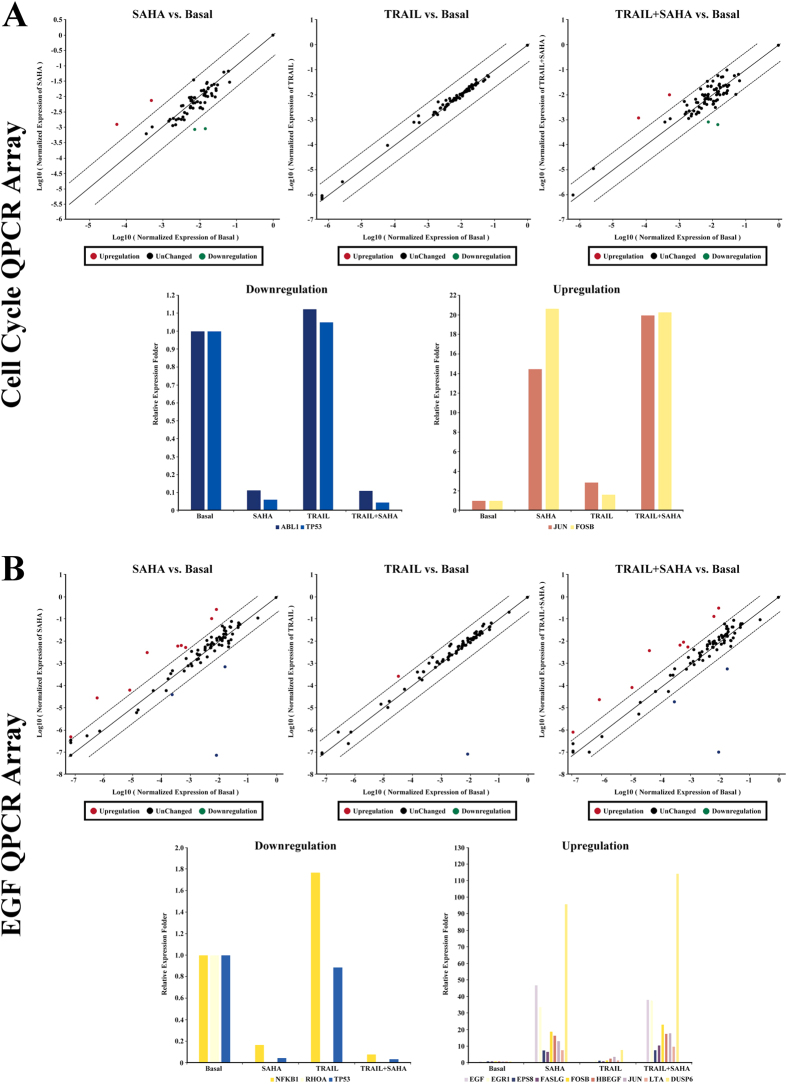
Q-PCR Array. (**A**) Human qPCR arrays for cell cycle related gene were employed to validate the mRNA expression for MCF-7 cells treatment with SAHA and TRAIL. Data normalization was based on correcting all *C*_t_ values for the average *C*_t_ values of GAPDH gene present on the array. Scatter plot and multigroup plot analysis were used respectively to show the data. Three independent biological replicates were performed. (**B**) Human qPCR arrays for EGF/PDGF signaling related gene were employed to validate the mRNA expression for MCF-7 cells treatment with SAHA and TRAIL. Data normalization was based on correcting all *C*_t_ values for the average *C*_t_ values of GAPDH gene present on the array. Scatter plot and multigroup plot analysis were used respectively to show the data. Three independent biological replicates were performed.

[Table t1] Furthermore, we sought to investigate the potential involvement of signaling mechanisms in proliferation and inhibition, as well as apoptosis induction by SAHA and TRAIL for breast cancer cells. We used quantitative PCR arrays to determine the expression levels of 168 genes, which included the regulation of apoptosis, cell cycle, growth factors and their receptors involved in signaling downstream of the growth factors, the genes targeted by growth factor-mediated stimulation, members of the MAP kinase pathway, the AKT/PI-3K pathway, the STATs pathway, and other downstream signaling cascades. The results showed 5-fold or greater changes of mRNAs as a result of treatment with SAHA and TRAIL, listed in Tables 1 and 2. We found pronounced and significant changes of WT1 and PDGFRA levels in MDA-MB-231 cells (Fig. 7A,B), FOSB, TP53, EGF, EGR1, DUSP6 level in MCF-7 cells (Fig. 8A,B), and RHOA level in both cell lines (Figs 7B and 8B). Importantly, the PDGFRA and DUSP6 levels were increased by over 50-fold in MDA-MB-231 and MCF-7 cell line, respectively. RHOA level decreased by more than 70,000-fold in both cell lines after treatment with SAHA and TRAIL. There were also significantly lower levels of TP53 in both cell lines with SAHA treatment alone and in combination with TRAIL, while no significant change was observed in the TRAIL treatment alone. For CDKN1A, SAHA or TRAIL treatment could elevate its mRNA expression, and the combination of treatments showed a synergistic effect of upregulation of CDKN1A in MDA-MB-231 cells. Since we only listed genes with 5-fold or greater change in mRNAs in Tables 1 and 2, CDKN1A was not included in Table 2, which was upregulated 4.84 and 4.97 times upon treatment with SAHA alone or the combinatorial treatment of SAHA and TRAIL in MCF-7 cells.

In addition, we found that SAHA treatment caused significant up-regulation or down-regulation of genes closely related to cell cycle regulation in MDA-MB-231 cell, including CCNA2, CCNB2, CDK6, CDKL1, RAD21, RB1, WEE1 and WT1. In MCF-7 cells, SAHA had more positive effects on the induction of apoptosis, with major changes in the expression of apoptotic genes including JUN, FOSB, NFKB1, EGF, EGR1, LTA, and DUSP6. Furthermore, several genes associated with cell cycle regulation were found to be suppressed by TRAIL in MDA-MB-231 cell, whereas no differences in mRNA expression was seen between vehicle and TRAIL treatment in MCF-7 cells.

**Figure 9:**
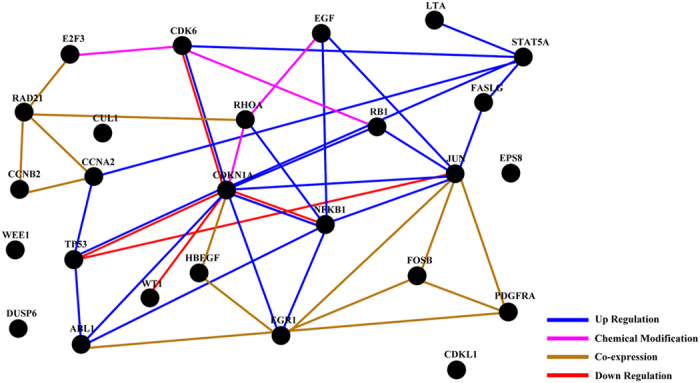
The relationships of genes through net-walking using GNCPro. GNCPro, free online software developed and maintained by SABiosciences, is an *in silico* research tool for collating gene and pathway interactions. The interactions among a group of genes are represented graphically and are interactive.

Finally, we assessed the relationships of 26 genes through net-walking using GNCPro^42^ (Fig. 9). CDKN1A is correlated with the most number of genes in this panel of 26 genes. Other interactive genes include JUN, EGR1, NFKB1, STAT5A, CDK6, TP53, RB1.

## Discussion

In this report, we first evaluated the effects exerted by SAHA in a triple negative breast cancer cell model, MDA-MB-231 and an ER+ MCF-7 cell lines. We used RTCA, which is an impedance-based technology that can be used for label-free and real-time monitoring of cell properties, such as cell adherence, proliferation, migration and cytotoxicity. RTCA is also a powerful and reliable tool that can be used in drug discovery for toxicity and pharmacology studies^43^,^44^,^45^. We found that SAHA treatment induced a time- and dose-dependent decrease in CI values in both cell lines. These results indicate that SAHA can be used as an agent in combinatorial treatment of both triple-negative and ER positive breast cancer cancers. Early study indicated that HDAC inhibitors such as trichostatin A, valproic acid, and SAHA inhibited proliferation of MCF-7 cells. When used in combination with tamoxifen, SAHA inhibited proliferation more potently than with either agent alone^46^. More recently, results from two published articles revealed that SAHA, together with Olaparib, a poly (ADP-ribose) polymerase (PARP) inhibitor^47^ or the naturally occurring sesquiterpene lactone Parthenolide (PN)^48^, were effective in inhibiting triple-negative breast cancer cell proliferation.

Unlike SAHA, TRAIL sensitivity varied with the phenotype of the breast cancer cell lines. Triple-negative breast cancer cells were very sensitive to TRAIL-induced apoptosis; in contrast, ER positive cells were resistant to TRAIL-induced apoptosis across a wide range of doses^49^. Previous studies also reveal that MDA-MB-231 cells exhibit a reduced sensitivity to TRAIL, but treatment with HDAC inhibitors restore cell sensitivity to TRAIL^40^,^41^,^50^. Our results showed that TRAIL or SAHA alone had only modest effect on reducing cell viability and cell number, but combined TRAIL and SAHA treatment significantly decreased cell viability and cell number in MDA-MB-231 and MCF-7 cells. In addition, TRAIL plus SAHA treatment significantly increased apoptotic cells. Our results confirmed the synergistic effect of SAHA and TRAIL in inducing apoptosis in both ER+ and ER- breast cancer cells.

Our results suggest that SAHA or SAHA plus TRAIL treatment plays a major role in the cell cycle G0/G1 phase, in the suppression of S phase, and consequently in the inhibition of proliferation and in inducing cell apoptosis in breast cancer cells. Treatment with SAHA caused a significant increase of cells in the G0/G1 phase and a decrease of cell numbers in the S phase in both breast cancer cell lines, whereas a much small increase of cells in G0/G1 phase was observed after treatment with TRAIL. The combination treatment of SAHA and TRAIL further increased the cells confined in the G0/G1 phase and marked decrease of cells in S phase in both cell lines. Interestingly, we also observed distinct morphological changes of cancer cells after treatment with TRAIL or SAHA alone. In MDA-MB-231, apoptotic morphology of cells was seen at 12 hrs after SAHA treatment, but not until 24 hrs after TRAIL treatment. Combination treatment of TRAIL and SAHA increased apoptotic cells to 50% of total at 24 hrs. In MCF-7, while SAHA alone was also seen as more effective than TRAIL alone in inducing apoptosis, combination treatment induced apoptosis at an earlier time (12 hrs). Additionally, combination treatment of breast cancer cells with TRAIL and SAHA caused detachment of cells followed by anoikis, a form of apoptosis which occurs after cell detachment^51^. Anoikis is induced upon cell detachment from extracellular matrix and anoikis-resistance contributes to metastasis allowing cancer cells to survive in the blood stream and invade distant sites^52^,^53^.

It has been reported that simultaneous administration of SAHA and TRAIL significantly upregulated the TRAIL death receptors DR4/DR5 and downregulated the anti-apoptotic members of the BCL-2 family in several tumor cell lines^54^,^55^,^56^,^57^. To better understand the molecular events that lead to the synergistic interaction of SAHA and TRAIL, we determined apoptosis-related molecule, BCL-2-associated X protein (BAX), Bcl-2, TRAIL related DR4, DR5 protein and mRNA expression using antibody array and real time PCR. BAX, the first BCL-2 interaction partner, regulates the critical balance between cellular life and death^58^,^59^. In contrast to BCL-2, BAX promotes, rather than blocks cell death after a stress stimulus^59^. We observed that combined TRAIL and SAHA treatments apparently increased Bax protein, Caspase-3 and CDKN1A in MDA-MB-231 cells and only Bax protein in MCF-7 than TRAIL or SAHA alone. These protein changes may explain why combined TRAIL and SAHA treatments are more effective in MDA-MB-231 than that in MCF-7 cells. We showed that SAHA inhibited Bcl-2 mRNA expression in MDA-MB-231 cell line. TRAIL or SAHA alone, as well as combined TRAIL and SAHA treatment significantly induced Bax mRNA expression in both of cell lines, and they also inhibited Bcl-2 mRNA expression in MDA-MB-231 cell line. Previous work showed inconsistent observation towards effects of SAHA and DR4, DR5 expression in breast cancer cell lines. Butler *et al*. observed that SAHA increased cell surface expression of DR5 but not DR4 in MDA-MB-231 cell lines^41^. However, Lauricella *et al*. observed that both of DR4 and DR5 were unmodified by SAHA in either MDA-MB-231 or MCF-7 cells^51^. We found that SAHA dramatically induced DR5 but not DR4 mRNA expression in both of MDA-MB-231 and MCF-7 cell lines. TRAIL alone and combined TRAIL and SAHA treatment also induced DR5 expression in both MDA-MB-231 and MCF-7 cell lines. A number of studies have demonstrated that both SAHA^41^,^48^,^51^,^60^,^61^ and TRAIL^62^,^63^,^64^ were able to activate Caspase-3 in breast cancer cell lines. We found that SAHA alone significantly induced Caspase-3 expression in MDA-MB-231 but not in MCF-7 cells. TRAIL alone and combined TRAIL and SAHA treatment also significantly induced Caspase-3 in MDA-MB-231 cells. Since MCF-7 cells lack Caspase-3 which was reported previously^62^,^65^, the very weak signal in the location of Caspase-3 in MCF-7 cells was likely unspecific. Cyclin-dependent kinase inhibitor 1A (CDKN1A) or p21WAF1/CIP1 was reported to be activated independent of p53 by SAHA^66^. Overexpression of CDKN1A may inhibit TRAIL death receptor DR4 dependent proximal caspase cleavage^67^. Our results demonstrated that TRAIL alone inhibited CDKN1A expression in MDA-MB-231 cells but induced CDKN1A expression in MCF-7 cells. However, combined TRAIL and SAHA treatment significantly induced CDKN1A expression, accompanied by decreased Phospho P53 (s46) expression in both cell lines.

We further determined the potential involvement of signaling mechanisms underlying the effects of SAHA and TRAIL for breast cancer cells using quantitative PCR arrays. The 168 candidate genes that were examined included apoptosis, cell cycle, growth factors and their receptors, as well as downstream signaling cascades. Wilms’ tumor 1 (WT1), reported to correlate with higher histological grades, ER-negative and basal-like and ERBB2 molecular subtypes in breast cancer^68^, was significantly downregulated in MDA-MB-231 cells upon treatment with SAHA or SAHA + TRAIL. The platelet-derived growth factor receptor α (PDGFRA), known to be associated with tumor aggressiveness in lymph node-negative breast cancer patients who had not received adjuvant systemic therapy^69^, after treatment of SAHA or SAHA + TRAIL was significantly upregulated MDA-MB-231 cells. In MCF-7 cells, genes that had over 5-fold changes upon treatment of SAHA and TRAIL alone or in combination include FosB, a member of the AP-1 family of transcription factors as important regulators of cell proliferation and differentiation and highly expressed in normal mammary epithelia but down-regulated in poorly differentiated breast cancer^70^, TP53, EGF, EGR1, and DUSP6. RhoA, one of the more extensively studied members of the Rho family of small GTPase, participate in tumor cell migration and invasion^71^, which was reported to be associated with progression in invasive breast duct carcinoma^72^ was most prominently downregulated in both cell lines. In addition, cell cycle related genes, such as CCNA2, CCNB2, CDK6, CDKL1, RAD21, RB1, WEE1 were also found to differentially expressed as a result of treatment in MDA-MB-231 cells, while expression of JUN, FOSB, NFKB1, EGF, EGR1, LTA genes were significantly altered in MCF-7 cells. The relationships of the 26 identified genes were further accessed through net-walking using GNCPro^42^. As shown in Fig. 9, most of the genes are related to each other, with the exception of WEE1, DUSP6, EPS8 and CDKL1. CDKN1A is identified as the most interactive gene in the group. Other active genes include JUN, EGR1, NFKB1, STAT5A, CDK6, TP53, RB1. These active genes appear to be involved in the molecular mechanisms of SAHA or TRAIL action in breast cancer cells. Our results suggest that the combinatorial treatment of SAHA and TRAIL may target multiple pathways. The exact sequence of signaling events induced by the treatment SAHA and TRAIL for breast cancer cells requires further clarification in future studies.

In conclusion, we investigated comprehensively the distinct effects of SAHA or TRAIL alone and in combination on the proliferation, cell viability, apoptosis, cell cycle distribution and morphological changes of MDA-MB-231 and MCF-7 cells. We observed that the different effects of SAHA or TRAIL alone and in combination on the expression of a number of apoptosis-related molecules, cell cycle, growth factors and their receptors in cancer cells. Our results demonstrated that the combinatorial treatment of SAHA and TRAIL may target multiple pathways and could serve as an effective therapeutic strategy against breast cancer. An improved understanding of the molecular mechanisms may facilitate either SAHA or TRAIL targeted use and the selection of suitable combinations.

## Materials and Methods

### Cell lines and Reagents

Human MDA-MB-231 and MCF-7 cells were purchased from American Type Culture Collection (ATCC) (Manassas, VA). Leibovitz’s L-15 medium, RPMI-1640 medium, Fetal Bovine Serum (FBS) and Penicillin-streptomycin Cocktails were obtained from Thermo Scientific (Rockford, IL). Suberanilohydroxamic acid (SAHA) was purchased from Sigma-Aldrich (St. Louis, MO). Recombinant human TRAIL/Apo2 Ligand was from Peprotech (Rocky Hill, NJ). Muse Cell Cycle kit, Muse Annexin & Dead Cell kit, and Muse Count & Viability kit were from Millipore (Darmstadt, Germany). Human Apoptosis Antibody Array kit was purchased from R&D Systems (Minneapolis, MN). High Pure RNA Isolation kit, Annexin-V-FLUOS staining kit and Transcriptor First Strand cDNA Synthesis kit were obtained from Roche Diagnostics GmbH (Mannheim, Germany). Exprofile Human Cell Cycle Tox and Cancer Related Gene qPCR Array kit and Exprofile Human EGF/PDGF Signaling Related Gene qPCR Array kit were obtained from Genecopoeia (Rockville, MD). Power SYBR Green PCR Master mix, Calcein-AM dye, RIPA Cell Lysis buffer and BCA Protein Assay kit were from Life Technologies (Austin, TX). CellTiter 96AQueous One Solution Cell Proliferation Assay kit was obtained from Promega (Madison, WI). Protease inhibitor and other chemicals were purchased from Sigma-Aldrich (St. Louis, MO).

### Cell culture

For all experiments, triplicate wells, tubes and reactions were run for each treatment and trials repeated at least three times with different cell preparations. The human breast cancer cell line MDA-MB-231 and MCF-7 were grown with Leibovitz’s L-15 medium and RPMI-1640 medium respectively, 15% fetal bovine serum (FBS), 100 units/ml penicillin and 100 μg/ml streptomycin were supplemented with the medium. For treatments, cells were seeded on uncoated flat-bottomed plastic plates (cell densities of 5.0 × 10^5^/well for 6 well plates, 1.0 × 10^4^/well for 96 well plates). In other sets of experiments, cells were cultured at a density of 1 × 10^6^ cells/35 mm or 2.0 × 10^7^/60 mm tissue culture dish. Semi-confluent cells were starved for 24 hours in basal medium (with DMSO) without FBS and treated with different compounds.

### Real-time monitoring the cell index (CI) assays

1.0 × 10^4^/well of MDA-MB-231 or MCF-7 cells was added into E-plate 16 in duplicates and xCELLigence Real-time Cell Analyzer (RTCA) DP system was used to monitor cell kinetics across microelectronic sensors integrated into the bottom. The cells were starved without FBS and incubated with medium containing SAHA (0, 0.5, 1, 2, 5, 10, 20 and 50 μM) or TRAIL (0, 5, 10, 20, 30, 40, 50, 100 ng/ml). The same concentrations of DMSO were used as control. The assay was monitored every 60 minutes for 120 hours. For quantification, the cell index (CI) values at indicated time points were graphically represented at least three independent measurements.

To confirm the results of RTCA, 1.0 × 10^4^/well of MDA-MB-231 or MCF-7 cells was loaded into 96 well plates. Subsequently, the cells were starved without FBS for 24 hours, and the medium in each well was replaced with medium containing various concentrations of SAHA or TRAIL as described above. The same concentrations of DMSO were used as control. For an additional 48 hours incubation, the culture supernatants were used to assess drug dose-response effects using the CellTiter 96 Aqueous One Solution Cell Proliferation Assay (Promega) according to the manufacturer’s protocol. The spectrophotometric absorbance of each sample was measured at 490 nm using TECAN spectora. The dose-response effect was defined by plotting the corrected absorbance versus concentration of drug.

### Cell Viability, Apoptosis and Cell Cycle assay

MDA-MB-231 cell was plated in 6 well plates at a density of 5 × 10^5^ cells per well. After synchronization with 5 μM DMSO (basal medium) without FBS for 24 hours, the cells were incubated in complete culture medium containing 5 μM SAHA or combining with 50 ng/ml TRAIL for 48 hours. MCF-7 cell was also plated in 6 well plates at a density of 5 × 10^5^ cells per well. After synchronization with 5 μM DMSO (basal medium) without FBS for 24 hours, the cells were incubated in complete culture medium containing 5 μM SAHA or combining with 100 ng/ml TRAIL for 24 hours.

Cell viability assay was performed using the Muse Count & Viability reagent (Millipore) following the manufacturer’s protocols. Following trypsinization, 2 × 10^5^ of harvested cells (50 ul cell suspension) were added with 450 ul Count & Viability reagent (Millipore). The results were obtained with Muse Count & Viability software module, and the statistics showed the percentage of viable cells. For the apoptotic assay, 1 × 10^6^ of cells were transferred in suspension to a new tube and incubated with 100 μl of Muse Annexin V & Dead Cell reagent (Millipore) for 20 minutes at room temperature. Muse Cell Analyzer (Millipore) determined apoptosis, and the statistics showed the percentages of the cells represented by alive, apoptosis and dead population. For cell cycle assay, 5 × 10^5^ cells were plated per well into a 6-well plate. After treatment with 5 μM SAHA or combining with 100 ng/ml TRAIL as described above, 10 μl PI were added to the cell suspension. Samples were filtered through 200-mesh filters and then analyzed on BD FACS Calibur flow cytometer. The DNA content of the cells was analyzed for the percentage of cells in G_1_, S and G_2_/M phase.

### Time-lapse live cell imaging acquisition

For our experiments, Nalge-Nunc “Lab-Tek^®^” coverglass system (Rochester, NY) was used to culture cells. Briefly, a density of 5 × 10^5^ cells was loaded in the each well of 4-chamber system. SAHA, TRAIL alone and combination treatments were initiated on experimental day 3 after starvation and aggregate formation. Phase contrast microscopic images of cells were obtained using the BioStation IM-Q (Nikon Corporation, Tokyo, Japan). BioStation IM-Q is an automatic cell maintenance system with a microscope and camera that enables scheduled automatic image acquisition. Image acquisition timing was set to every 12 hour (magnification = 20x) and time points were designated as time 0, 12, 24, 36 and 48, indicating each of the 12 hour imaging intervals.

### Apoptotic fluorescence detection

Annexin-V-FLUOS staining kit was used for apoptosis detection following the manufacturer’s instruction. Briefly, 5 × 10^5^ cells were seeded onto each well of 12 well plates and allowed to adhere overnight to be treated with desired concentrations of SAHA and TRAIL for 0, 12, 24, 36 and 48 hours. Cells were added 100 μL Annexin-V-FLUOS labeling solution and incubated for 15 minutes at 15 °C–20 °C and immediately analyzed by fluorescence microscopy.

### RNA extraction and Real-time PCR

RNA was extracted from cells using high pure RNA isolation kit following the manufacturer’s protocols. First-strand cDNA was synthesized from total RNA using transcriptor first strand cDNA synthesis kit. The cDNA was used as a template in real-time PCR reactions with Power SYBR Green PCR Master mix and was run on an Applied Biosystems 7500 real-time PCR system. The 25 μl real-time quantitative PCR reaction mixture consisted of 1x SYBR Green Supermix, 0.25 mmol/L forward and reverse primers, and 10 ng cDNA. The PCR conditions were 50 °C for 2 minutes, 95 °C for 2 minutes, followed by 40 cycles at 95 °C for 15 seconds and 60 °C for 1 minute. Relative gene expression quantifications were calculated according to the comparative Ct method using GAPDH as an endogenous control. Final results were determined by the 2^−△△Ct^ formula. Validated primers against human Bax mRNA (forward: 5′-GGGGACGAACTGGACAGTAA-3′; reverse:5′-CAGTTGAAGTTGCCGTCAGA-3′), human Bcl-2 mRNA (forward: 5′-TGTTGTTCAAACGGGATTCA-3′; reverse: 5′-GGCTGGGCACATTTACTGTT-3′), human TRAIL-DR4 mRNA (forward: 5′-GGAACTTTCCGGAATGACAA-3′; reverse: 5′-GTCACTCCAGGGCGTACAAT-3′), human TRAIL-DR5 mRNA (forward: 5′-ATTTCAGCCTCTTTCCAGCA-3′; reverse: 5′-CGGAACAAAACACACAATGC-3′), human GAPDH mRNA (forward: 5′-GAGTCAACGGATTTGGTCGT-3′; reverse: 5′-GACAAGCTTCCCGTTCTCAG-3′) were used in the experiment.

### Human apoptosis antibody array

The array is a rapid, sensitive tool to detect the relative levels of expression of apoptosis-related proteins simultaneously. The experiment was carried out in accordance with manufacturer’s instructions. First, approximately 1 × 10^7^ cells with SAHA and TRAIL treatment were solubilized in lysis buffer and centrifuged at 14000 g for 5 minutes. Protein concentrations of the resulting lysates were measured using a BCA protein assay kit. Next, each of antibody-coated array membranes was placed into the provided dish, and 200 ug of prepared cell lysates were added each well of the dish to incubate at 4 °C with gentle shaking overnight. The membranes were washed with wash buffer and then incubated with 1.5 ml lyophilized biotinylated antibodies for 1 hour on a rocking platform shaker. The mixture of biotin-conjugated antibodies was removed, and membranes were incubated with horseradish peroxidase -conjugated streptavidin for 30 minutes. After a final wash, membrane intensity was acquired using chemiluminescence and pixel densities can be analyzed using Gelpro Analyzer software (Media Cybernetics, Rockville, MD). Densities were measured as a percentage of the positive controls included on each membrane. After subtracting background signals and normalization to positive controls, comparison of signal intensities among array images can be used to determine relative differences in expression levels of each protein between groups.

### Q-PCR array

To validate relative results of mRNA expression, human qPCR arrays for cell cycle tox and cancer related gene and EGF/PDGF signaling related gene were employed. Briefly, total RNA was collected from cells using high pure RNA isolation kit with DNase I. RNA was quantified with a Nanodrop 2000 machine (Thermo Scientific). A total of 1 μg RNA was used for reverse transcription with transcriptor first strand cDNA synthesis kit. Q-PCR reactions were performed on a 7500 Real-time PCR system following manufacturer’s instructions. Data normalization was based on correcting all *C*_t_ values for the average *C*_t_ values of GAPDH gene present on the array. Three independent biological replicates were performed.

To analyze the PCR-array data, an MS-Excel sheet with macros was downloaded from the website (https://www.qiagen.com/us/products/genes%20and%20pathways/data-analysis-center-overview-page/), imported the *C*_t_ values into the template sheet, uploaded the template form and chose the correct analysis factors. Relative changes of gene expression in the array were calculated using the 2^−ΔΔCt^ (threshold cycle) method.

### Data analysis

Student’s *t*-test was used for data analysis. Data are presented as mean ± SEM. Values for *p* < 0.05 were considered statistically significant. The model included the main effects of treatments and replicates.

## Additional Information

**How to cite this article**: Zhou, W. *et al*. Synergistic effects of combined treatment with histone deacetylase inhibitor suberoylanilide hydroxamic acid and TRAIL on human breast cancer cells. *Sci. Rep*. **6**, 28004; doi: 10.1038/srep28004 (2016).

## Figures and Tables

**Table 1 t1:** The change levels of mRNAs expression encoding apoptosis, cell cycle and necessary pathways in MDA-MB-231 cells treatment with SAHA and TRAIL.

Gene Name	Description	Fold change in mRNA level		
		SAHA	TRAIL	SAHA TRAIL
ABL1 NM_005157	c-abl Oncogene 1	−5.89	−1.61	−5.01
CCNA2 NM_001237	Cyclin A2	−5.71	−4.77	−11.10
CCNB2 NM_004701	Cyclin B2	−7.01	−3.23	−9.25
CDK6 NM_001259	Cyclin-dependent kinase 6	−8.01	−3.40	−5.44
CDKL1 NM_004196	Cyclin-dependent kinase-like 1	−5.21	−3.76	−6.00
CDKN1A NM_000389	Cyclin-dependent kinase Inhibitor 1A (p21, Cip1)	5.10	4.76	10.85
CUL1 NM_003592	Cullin 1	−12.39	−2.00	−7.34
E2F3 NM_001949	E2F Transcription Factor 3	−5.80	−2.39	−5.45
EGF NM_001963	Epidermal Growth Factor	4.89	1.27	5.66
FASLG NM_000639	Fas Ligand	3.78	0.93	3.66
FOSB NM_006732	FBJ Murine Osteosarcoma Viral Oncogene Homolog B	5.73	−2.16	3.83
NFKB1 NM_003998	Nuclear Factor of kappa Light Polypeptide Gene Enhancer in B-cell 1	−16.37	−2.09	−12.29
PDGFRA NM_006206	Platelet-derived Growth Factor Receptor, alpha Polypeptide	69.93	−1.21	39.01
RAD21 NM_006265	RAD 21 Homolog	−2.31	−2.17	−3.12
RB1 NM_000321	Retinoblastoma 1	−5.18	−7.23	−5.11
RHOA NM_001664	RAS Homolog Gene Family, Member A	−4,236,340	−5,164,372	−1,184,666
STAT5A NM_003152	Signal Transducer and activitor of Transcription 5A	−5.69	−1.60	−5.69
TP53 NM_000546	Tumor Protein p53	−10.48	−1.88	−11.04
WEE1 NM_003390	Wee1 Homolog	−4.28	−3.40	−5.89
WT1 NM_000378	Wilms Tumor 1	−16.24	−2.62	−20.52

**Table 2:** The change levels of mRNAs expression encoding apoptosis, cell cycle and necessary pathways in MCF-7 cells treatment with SAHA and TRAIL.

Gene Name	Description	Fold change in mRNA level		
		SAHA	TRAIL	SAHA TRAIL
ABL1 NM_005157	c-abl Oncogene 1	−8.96	1.12	−9.02
DUSP6 NM_001946	Dual Specificity Phosphatase 6	115.91	8.19	178.13
EGF NM_001963	Epidermal Growth Factor	46.83	−2.47	38.18
EGR1 NM_001964	Early Growth Response 1	33.67	1.67	37.86
EPS8 NM_004447	Epidermal Growth Factor Receptor Pathway Substrate 8	7.61	1.59	7.79
FOSB NM_006732	FBJ Murine Osteosarcoma Viral Oncogene Homolog B	19.04	1.76	23.29
FASLG NM_000639	Fas Ligand	6.76	1.10	10.76
HBEGF NM_001945	Heparin-binding EGF-like Growth Factor	16.46	2.72	17.68
JUN NM_002228	Jun Proto-oncogene	14.45	2.88	19.96
LTA NM_000595	Lymphotoxin A	7.85	1.78	10.00
NFKB1 NM_003998	Nuclear Factor of kappa Light Polypeptide Gene Enhancer in B-cell 1	−5.94	1.77	−12.85
RHOA NM_001664	RAS Homolog Gene Family, Member A	−107,493	−97,879	−79,423
TP53 NM_000546	Tumor Protein p53	−16.36	1.05	−22.29
